# Differential regulated microRNA by wild type and mutant p53 in induced pluripotent stem cells

**DOI:** 10.1038/cddis.2016.419

**Published:** 2016-12-29

**Authors:** Francesca Grespi, Vivien Landré, Alina Molchadsky, Nicola Di Daniele, Luigi Tonino Marsella, Gerry Melino, Varda Rotter

**Affiliations:** 1 Department of Biology, University of Padua, Padua, Italy; 2 Medical Research Council, Toxicology Unit, Leicester University, Hodgkin Building, Leicester, UK; 3 Department of Molecular Cell Biology, Weizmann Institute of Science, Rehovot, Israel; 4 Faculty of Medicine and Surgery, University of Rome 'Tor Vergata', Rome, Italy

## Abstract

The tumour suppressor p53 plays an important role in somatic cell reprogramming. While wild-type p53 reduces reprogramming efficiency, mutant p53 exerts a gain of function activity that leads to increased reprogramming efficiency. Furthermore, induced pluripotent stem cells expressing mutant p53 lose their pluripotency *in vivo* and form malignant tumours when injected in mice. It is therefore of great interest to identify targets of p53 (wild type and mutant) that are responsible for this phenotype during reprogramming, as these could be exploited for therapeutic use, that is, formation of induced pluripotent stem cells with high reprogramming efficiency, but no oncogenic potential. Here we studied the transcriptional changes of microRNA in a series of mouse embryonic fibroblasts that have undergone transition to induced pluripotent stem cells with wild type, knock out or mutant p53 status in order to identify microRNAs whose expression during reprogramming is dependent on p53. We identified a number of microRNAs, with known functions in differentiation and carcinogenesis, the expression of which was dependent on the p53 status of the cells. Furthermore, we detected several uncharacterised microRNAs that were regulated differentially in the different p53 backgrounds, suggesting a novel role of these microRNAs in reprogramming and pluripotency.

The tumour suppressor p53 is the most frequently mutated or deregulated gene in human cancers.^1, 2, 3, 4, 5, 6, 7^ Often referred to as the guardian of the genome, its role in protecting the cell from accumulation of DNA damage by inducing DNA repair or cell death is well-studied.^8, 9, 10, 11, 12^ However, p53 has also been implicated in a vast variety of other cell pathways, including metabolism,^13^ autophagy,^14, 15^ mitochondrial function^16, 17, 18^ and also cell differentiation and pluripotency.^19, 20^ Interestingly, p53 mutations, in addition to disrupting the protein's wild-type function, result in additional activities that lead to increased tumour malignancy, usually referred to as gain of function (GOF).^21, 22^

Recently, p53 is emerging as a key regulator in the process of reprogramming from somatic to induced pluripotent stem (iPS) cells as well as being involved in stem cell maintenance.^23, 24, 25, 26, 27, 28, 29, 30^ Stem cells are characterised by high genomic stability, which is crucial to minimise tumorigenesis following stem cell expansion.^31, 32, 33^ p53 is an important factor that protects this genomic integrity and has the ability to counteract somatic reprogramming by inducing cell cycle arrest and apoptosis.^23, 25, 26, 34, 35, 36^ In contrast to somatic cells, p53 does not induce apoptosis in embryonic stem cells (ESCs) following DNA damage, but promotes differentiation of ESC by several mechanisms including transcriptional repression of the pluripotency factors Nanog and Oct4.^37, 38, 39, 40^ After differentiation p53 activates the expression of genes that lead to cell death or senescence by classical p53 pathways. Thus, p53 plays an important role in maintaining a pool of stem cells with an intact genome and moreover prevents of reprogramming cells with faulty genome.^27^

We have previously studied the reprogramming efficiency of a series of MEFs with different p53 status, that is, p53 wt, p53 knock out (KO) and mutant p53R172H cells.^27^ p53R172H (R175H in human) is a conformational mutant that results in a misfolded p53 protein. This study showed that p53 depletion or the expression mutant p53 increases reprogramming efficiency.^27^ However, cells expressing p53R172H in addition to their augmented pluripotency *in vitro* exhibited carcinogenic potential *in vivo*. When injected into nude mice, p53R172H expressing iPS cells lost their differentiation capacity and gave rise to aggressive sarcomas, while p53 KO iPS cells maintained pluripotency and led to the formation of benign teratomas, thus displaying a novel GOF for mutant p53.^27^

It is of great interest to generate iPS cells with a high reprogramming efficiency, but low tumorigenic potential for therapeutic use. As p53 was shown to be important in both reprogramming and maintaining genomic integrity of iPS cell, it provides an interesting target for manipulation of the reprogramming pathway. It is thus of interest to dissect the mechanisms and players regulated by p53 in these pathways. In addition to controlling the expression of protein coding genes, p53 was shown to control the transcription of a number of microRNAs (miRNAs). Expression of miRNAs is altered in many pathological conditions including cancer, where different miRNAs exhibit oncogenic and tumour suppressive properties. Moreover, miRNAs are key regulators of development; for example, miR-34a is fundamental for neuronal and muscle differentiation,^41, 42, 43^ but also influence reprogramming of stem cells and the maintenance of an undifferentiated cellular stage.^44, 45^

In this study, we set out to examine miRNAs that are differentially regulated in cells during reprogramming depending on their p53 status, aiming to identify miRNAs that play a role in this process and that could be directly targeted to help optimise iPS cells. This would allow the generation of cells that have intact p53, which protects their genomic integrity, but at the same time exhibit high reprogramming efficiency. To this end, we performed a microarray screening of miRNA expression before and after three factors driven reprogramming of wt, KO and mutant p53 cells and identified several miRNAs whose expression is dependent on the p53 status of the cell.

## Results

### Identification of microRNAs that are modulated during the MEF to iPS cell transition depending on cell's p53 status

To identify miRNAs that are targeted by either wt or mutant p53 during reprogramming, we performed microarray analysis on a series of iPS cells and their parental MEFs with different p53 (wt, p53, KO and p53R172H). The MEFs were reprogrammed by introduction of Oct4, Sox2, Klf4 as described in our previous study by Sarig *et al.*^27^

All Cq values above 32 were considered noise background and excluded from the analysis. The results for a given sample were normalised by the geometric mean of the relative quantities of all targets expressed in the same sample (global mean procedure). Targets that were up- or downregulated more than twofold were considered to be changed in the analysis.

**Figure 1:**
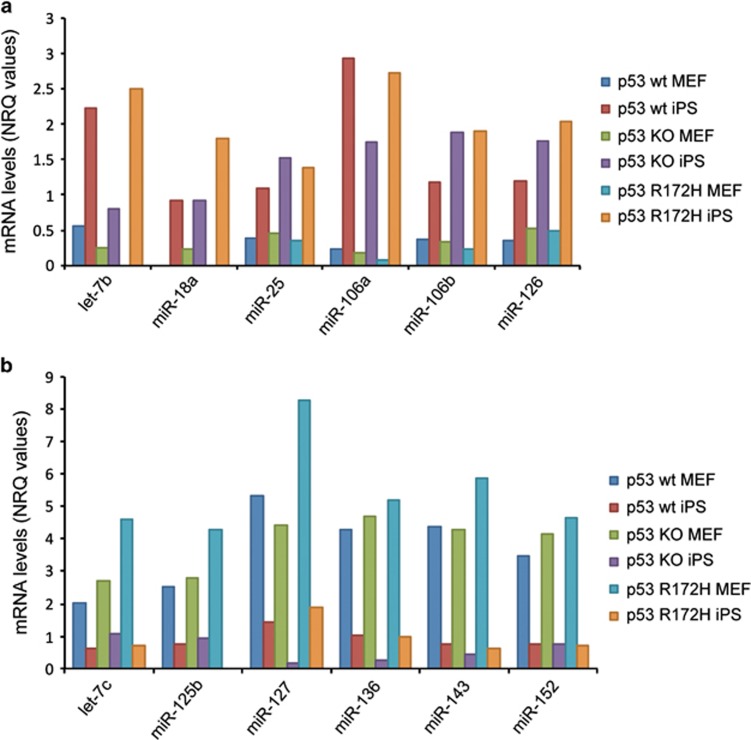
A number of miRNAs involved in differentiation are up/downregulated during the MEF to iPS cell transition independent of the cell's p53 status. MiRNAs levels were examined upon transition from MEFs to iPS cell using microarray analysis. Selected miRNAs that were induced (**a**) or decreased (**b**) in all genetic backgrounds, that is, p53 wt, p53R172H and p53 KO (NRQ=normalised relative quantities)

In a first step we looked at the miRNAs that were up- or downregulated in all three conditions during reprogramming (Figure 1). A high number of the detected miRNAs are known to play a role in stemness and differentiation, that is, let7 family, miR-125b, miR-126, miR-136, miR-143, miR-145, miR-152, suggesting that we indeed identified miRNAs that are important in reprogramming using our experimental set up.

**Figure 2:**
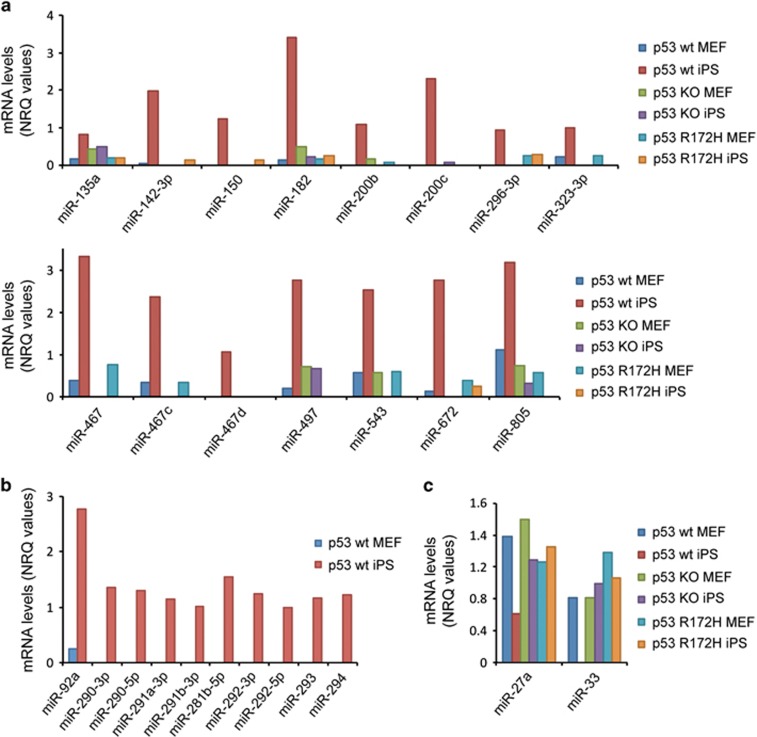
Several miRNAs are regulated exclusively in p53 wt cells. MiRNAs levels were examined upon transition from MEFs to iPS cells using microarray analysis. Selected miRNAs that were induced exclusively in p53 wt cells (**a**), only detected in p53 wt cells (**b**) and downregulated in p53 wt cells (**c**) are shown

Next, to study the role of p53, both wt and mutant, during reprogramming, we were interested in miRNAs that were specifically regulated depending on the cell's p53 status (see Supplementary Table S1 for summary of relevant miRNAs identified). Figure 2a shows those that were upregulated specifically by wt p53 in iPS cells, while not significantly changed in the other conditions. Of these, miR-200c was previously shown to be a transcriptional target of p53.^46, 47^ Furthermore, several of these miRNAs have been implicated in both stemness and cancer (Supplementary Table S1). MiR-182, for example, is an important factor in the development of the inner ear and retina, T-cell development and osteogenesis and has also been implicated in cancer development and metastasis,^48^ while miR-497 is a tumour suppressor that has been shown to induce quiescence in skeletal muscle stem cells.^49, 50, 51^

Additionally, we identified a number of small non-coding RNAs that were only detectable in iPS cells bearing functional p53 (Figure 2b), most of which have not yet been characterised. Furthermore, we identified two miRNAs, miR-27a, see also refs 52, 53, 54, and miR-33 that were downregulated following reprogramming exclusively in p53 wt cells (Figure 2c).

**Figure 3:**
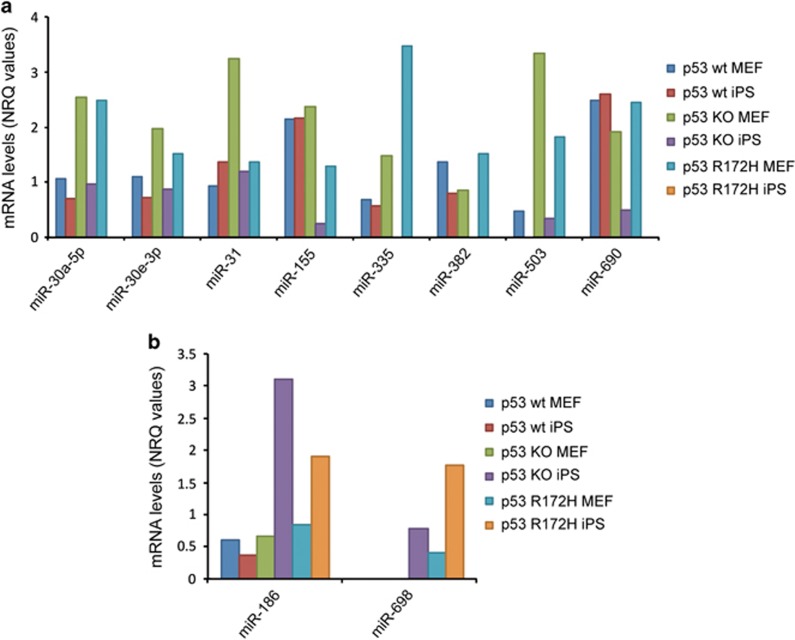
Levels of some miRNAs change solely in p53R172H and p53 KO cells during the MEF to iPS cell transition. Analysis of miRNAs levels upon transition from MEFs to iPS cells highlighted miRNAs that were regulated differentially depending on the cell's p53 status. Here miRNAs that were induced (**a**) or decreased (**b**) in p53R172H and p53 KO cells, while unchanged in a p53 wt background are shown

Both p53 KO and p53 mutant cells displayed increased reprogramming efficiency compared with wt p53, notably p53 mutant cells underwent reprogramming more effectively than p53 KO cells. We were thus interested in hits that were regulated in these conditions and found several miRNA that are downregulated (Figure 3a) or upregulated (Figure 3b) in p53 KO and mutant cells, but not changed in p53 wt cells. For example, we found that p53 KO and p53 mutant iPS cells induced the expression of miR-186. Furthermore, a high number of miRNAs that were downregulated in p53 compromised iPS cells convey tumour suppressive functions, that is, miR-30a-5p,^55^ miR-31,^56, 57^ miR-335,^58^ miR-382^59^ and miR-503.^60, 61^

### Mutant p53 regulates a specific pool of microRNAs during reprogramming that might be linked to its GOF activity

**Figure 4:**
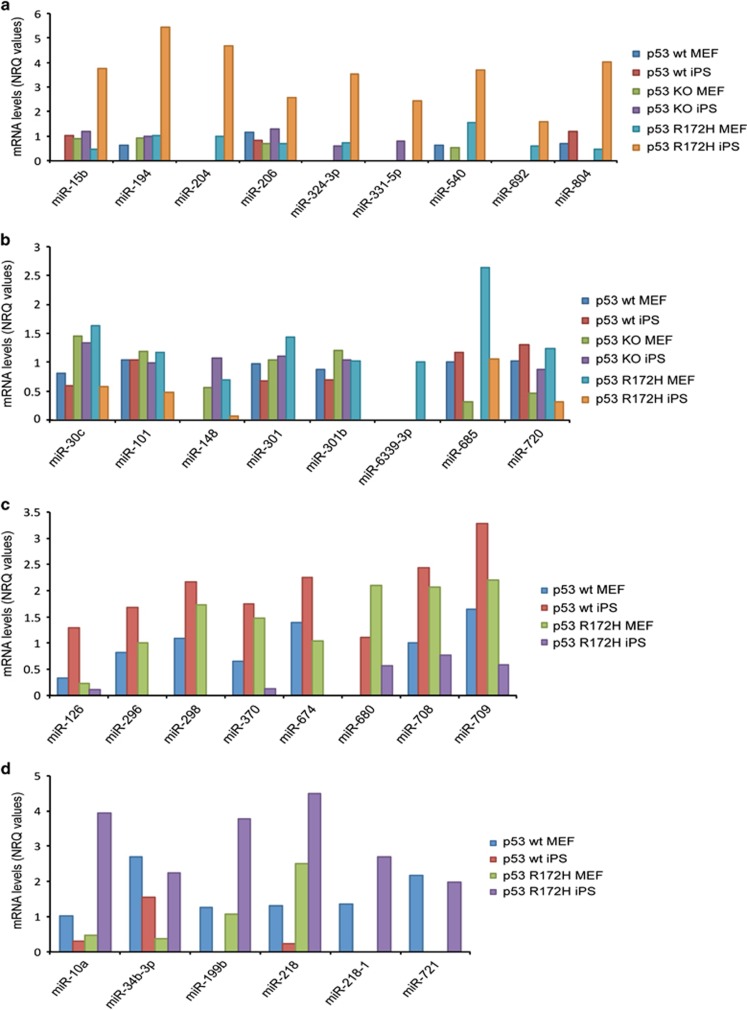
Complex regulation of miRNAs during the induced transition from MEF to iPS cells depending on p53. Analysis of miRNAs levels upon transition from MEFs to iPS cell revealed a group of miRNAs that was upregulated (**a**) or downregulated (**b**) only in p53R172H iPS cell, while unchanged in the other groups, and miRNAs whose expression was induced in p53 wt while reduced in KO or p53 mutant cells (**c**) or vice versa (**d**)

In previous studies we demonstrated a GOF activity of mutant p53 in reprogramming, manifested not only by more efficient process, but also by acquiring in cells with tumorigenic properties.^27^ We were therefore particularly interested to identify miRNA that are specifically modulated in p53R172H cells during MEF to iPS cell transition. We identified several miRNAs that are strongly upregulated (Figure 4a) or downregulated (Figure 4b) upon reprogramming in p53R172H cells, while unchanged or less strongly regulated in the other samples. As mutant p53 cells have a high reprogramming efficiency, these miRNAs could facilitate the reprograming process and it would be interesting to study their role in more detail. Indeed several of the miRNA regulated by mutant p53 during transition from MEF to iPS cells have been implicated with stemness as well as carcinogenesis (Supplementary Table S1). For example, miR-194 and miR-206 were previously found to be involved in osteoblast^62^ and muscle differentiation,^63^ respectively, while miR-101, whose expression was decreased in p53R172H iPS cells, is a well-characterised tumour suppressor that inhibits tumour growth and metastasis.^64, 65, 66, 67, 68^

### Reversely regulated microRNAs in p53 wt and p53 mutant cells

Interestingly, we identified several miRNAs whose expression was opposite in p53 wt and p53 mutant cells, that is, increased expression during reprogramming in wt cells, yet decreased in mutant cells or vice versa (Figures 4c and d). This suggests that these miRNAs are regulated by p53 during reprogramming by complex mechanisms. An example is miR-10a that on the one hand is known to promote the differentiation of human mesenchymal stem cells^69^ and on the other contributes to cancer development.^70, 71^ This miR is downregulated during reprogramming of p53 wt cells, but upregulated in cells expressing mutant p53. Mir-199b shows upregulation in p53R172H iPS. The same is true for miR-218, an miRNA that seems to acts as a tumour suppressor^72, 73^ and promotes stem cell differentiation.^74, 75, 76^ The tumour suppressive miR-708 and miR-126 on the other hand are upregulated during reprogramming in p53 wt cells and reduced in p53R172H cells.

### MicroRNAs specifically upregulated in WT p53 iPS cell encode for a p53 responsive element

To further characterise the miRNA that we identified as upregulated solely in p53 wt cells, we decided to screen for predicted responsive elements in the promoter region of those miRNAs that were induced upon reprogramming in the presence of wt p53 (Figure 2). We identified four miRNAs that encode a p53 responsive element with high matrix similarity in their promoter region (Supplementary Table S2). Of all the targets that were specifically upregulated during reprogramming of wt cells, only miR-142-3p lacked a predicted p53 responsive element in its promoter altogether.

Next, we investigated whether any of the miRNAs upregulated specifically in p53 wt cells are predicted to target genes known to be important for reprogramming and pluripotency. While we did not identify any of the key regulators of reprogramming, such as Nanog, Sox2, Oct4/3, ERK2 or *β*-catenin/Ctnnb1 as predicted targets, our predictive analysis highlighted factors, for example, N-Myc, Akt1/2/3 and Smad 2/2, involved in iPS cell generation and pluripotency that encode a responsive element in their 3′UTR for one or several of the miRNAs that we identified in our screen (Supplementary Table S3).

## Discussion

In this study we used our previously published iPS cell model^27^ to investigate miRNAs that are under control of p53, wild type or mutant during reprogramming. The fact that p53 mutant cells exhibit an increased reprogramming efficiency, while p53 wt iPS cells have high genomic stability and thereby low carcinogenic potential, makes this transcription factor and its targets of great interest in the aim to optimise pluripotent stem cell formation.

Using a microarray approach we were able to identify several miRNAs that are specifically regulated by p53 during the process of reprogramming. Interestingly, we did not only find miRNAs upregulated by wt p53, but also specifically by p53R172H. We have previously shown that this p53 mutant exhibits a GOF activity during reprogramming^27^ and it would be very interesting to further investigate whether these miRNAs are responsible for this phenotype. Among the miRNAs that are regulated by p53R172H during the MEF to iPS cell transition, several have known functions in stemness and differentiation, for example miR-186, miR-194 and miR-206. Additionally, several miRNAs that are known to exhibit tumour suppressive functions, like miR-30a-5p, miR-31, miR-335, miR-382 and miR-503, were downregulated in the p53R172H cells upon reprograming to iPS cells. This is in line with our earlier study that showed occurrence of malignant tumours after injection of iPS cells with p53R172H in mice.^27^ On the other hand, we also identified tumour suppressive miRNAs, like miR-218 that are upregulated in p53 mutant cells, and in the case of miR-218 downregulated in p53 wt cells. The roles of these miRs in the reprogrammed cells remains to be studied.

Interestingly, miR-15b that was markedly induced exclusively by mutant p53 during reprogramming. Mir-15b promotes osteoblast differentiation^77^ whilst it reduces invasion and metastasis^78, 79^ and its deletion was shown to promote B-cell malignancies;^80^ these distinct effects suggests a possible complex context-dependent function. Furthermore, we detected a decrease of miR-155 in both p53 KO and mutant cells during reprogramming, while its levels remained high in p53 wt cells, which is at variance with previous studies.^81,82^

Notably, we also identified miRNAs that are induced in p53 KO and p53 mutant iPS cells, but not in p53 wt iPS cells, suggesting that wt p53 inhibits their up-regulation during reprogramming. It remains to be investigated how these miRNAs effect differentiation and/or oncogenesis *in vivo*.

We identified several miRNAs that are increased specifically in p53 wt cells during reprogramming from MEF to iPS cells. These miRNAs could be involved in ensuring high genome stability of reprogrammed cells. MiR-709,^83^ for example, is upregulated specifically in the transition from p53 wt MEFs to iPS cells, while it is downregulated when p53 is not functional. Another interesting hit that is strongly upregulated in p53 wt iPS cells, but very low expressed in p53 KO and mutant iPS cells is miR-142-3p.^84,85^ We were unable to identify whether a p53 responsive element is its promoter, suggesting that p53 regulates the expression of this miRNA by an indirect mechanism.

Of note, our microarray screening did not highlight miR-34a as upregulated during reprogramming, as previously reported.^86, 87, 88^ We think this could be due to the fact that in the previous work, iPS cells were generated with four reprogramming factors (Oct4, Sox2, Klf4 and c-Myc), while in this study the iPS cells were generated using only three reprogramming factors (Oct4, Sox2 and Klf4) since c-Myc has been correlated with carcinogenesis of iPS cells *in vivo*.^89^

In conclusion, our study highlighted a group of miRNAs driven by wt and mutant p53 that might be important for reprogramming, tumorigenesis and loss of genomic integrity. While a high number of miRNAs identified in our screen have already been associated with cancer development or differentiation and stemness (Supplementary Table S1), others are less well characterised and provide interesting targets to study in more details. It would be of particular interest to dissect their role during reprogramming and how this is controlled by p53 in either its wild type or mutant form. Thus, further studies will be necessary to further validate these results and pinpoint the exact functions of these miRNAs during the formation of iPS cells.

## Materials and Methods

### Cell culture and iPS cell generation

Primary MEFs were prepared from wt, p53 mutant or p53 KO E13.5 embryos as reported previously.^27^ Briefly, MEFs were prepared from 13.5 days postcoitum embryos and maintained in DME, supplemented with 10% FCS, 1 mM sodium pyruvate, 2 mM l-glutamine, 0.1 mM nonessential amino acids, 0.1 mM *β*-mercaptoethanol and antibiotics. Reprogramming was induced using the three factor protocol (Oct4, Sox2, Klf4) as described previously.^27^

### RNA extraction and microarray analysis

RNA was extracted using Trizol (Invitrogen, USA), following manufacturer's instruction.

Microarray analysis was performed by Biogazelle (Gent, Belgium). All Cq values above 32 were excluded as noise background. The results for a given sample were normalised by the geometric mean of the relative quantities of all targets that are expressed in that sample (global mean normalisation procedure). Three biological samples (*n*=3) were collected and pooled together for technical reasons; these were analysed with three technical replicates, obtaining always a mean error lower than 5% of the measure. Because this variation derives from a technical measure, and not from a biological replicate, it has not been reported on the histograms. Therefore, we did not show any statistics on these qualitative results.

### Predictive analysis

P53 responsive element in microRNAs promoters were predicted using the programme MatInspector that allows identification of transcription factors binding sites in nucleotide sequences using a large library of weight matrices (Genomatix, Germany).

MicroRNAs targets were predicted by using the miRanda Software (microrna.org). Only target sites of conserved miRNAs and with good mirSVR score were taken into consideration.
