# Supplementary material for: Differential regulated microRNA by wild type and mutant p53 in induced pluripotent stem cells
Source: Cell Death Dis. 2016 Dec 29;7(12):e2567–. doi: 10.1038/cddis.2016.419 (PMC5260988; doi:10.1038/cddis.2016.419)
Supplement: Supplementary Tables [file cddis2016419x1.doc]

|  |  | | | **Known function in:** | |
| --- | --- | --- | --- | --- | --- |
|  | **p53 wt** | **p53 KO** | **p53^R172H^** | **Differen-tiation & Stemness** | **Cancer** |
| **miR-10a** | 🡻 | 🡹 | 🡹 | (1-4) | (5-11) |
| **miR-15b** |  |  | 🡹 | (12) | (13-17) |
| **miR-27a** | 🡻 |  |  | (18-24) | (25-31) |
| **miR-30a-5p** |  | 🡻 | 🡻 | (32) | (33-36) |
| **miR-30c** |  |  | 🡻 | (37) | (38-41) |
| **miR-30e-3p** |  | 🡻 | 🡻 | - | (42) |
| **miR-31** |  | 🡻 | 🡻 | (43-45) | (46-50) |
| **miR-33a** | 🡻 |  |  | - | (51-53) |
| **miR-34b-3p** | 🡻 |  | 🡹 | - | - |
| **miR-92a** | 🡹 |  |  | (54-56) | (57-59) |
| **miR-101** |  |  | 🡻 | - | (60-66) |
| **miR-126** | 🡹 | 🡹 | 🡻 | (67-72) | (73-77) |
| **miR-135a** | 🡹 |  |  | (78, 79) | (80-83) |
| **miR-142-3p** | 🡹 |  |  | (84, 85) | (86-88) |
| **miR-148b** |  |  | 🡻 | - | (89-92) |
| **miR-150** | 🡹 |  |  | (93-97) | (98-102) |
| **miR-155** |  | 🡻 | 🡻 | (103-105) | (106-109) |
| **miR-182** | 🡹 |  |  | (110, 111) | (111-117) |
| **miR-186** |  | 🡹 | 🡹 | - | (118-120) |
| **miR-194** |  |  | 🡹 | (121-123) | (124-127) |
| **miR-199b** | 🡻 | 🡻 | 🡹 | (128) | (129, 130) |
| **miR-200b** | 🡹 |  |  | (131) | (132-136) |
| **miR-200c** | 🡹 |  |  |  | (137, 138) |
| **miR-204** |  |  | 🡹 | (139) | (140-145) |
| **miR-206** |  |  | 🡹 | (146, 147) | (148, 149) |
| **miR-218** | 🡻 |  | 🡹 | (150-152) | (148, 149, 153, 154) |
| **miR-218-1** | 🡻 | 🡻 | 🡹 | - | (155) |
| **miR-290-3p** | 🡹 |  |  | - | (156) |
| **miR-290-5p** | 🡹 |  |  | - | (156) |
| **miR-291a-3p** | 🡹 |  |  | - | - |
| **miR-291b-3p** | 🡹 |  |  | - | - |
| **miR-291b-5p** | 🡹 |  |  | - | - |
| **miR-292-3p** | 🡹 |  |  | - | - |
| **miR-292-5p** | 🡹 |  |  | - | - |
| **miR-293** | 🡹 |  |  | - | - |
| **miR-294** | 🡹 |  |  |  | (157, 158) |
| **miR-296** | 🡹 |  |  | - | (159, 160) |
| **miR-296-3p** | 🡹 |  | 🡻 | - | (161, 162) |
| **miR-298** | 🡹 |  | 🡻 | - | (163) |
| **miR-301** |  |  | 🡻 | - | (164) |
| **miR-301b** |  |  | 🡻 | - | (165-167) |
| **miR-323-3p** | 🡹 |  |  | (168) | (169) |
| **miR-324-3p** |  |  | 🡹 | - | (170) |
| **miR-331-5p** |  |  | 🡹 | - | (171) |
| **miR-335** |  | 🡻 | 🡻 | (172, 173) | (174, 175) |
| **miR-339-3p** |  |  | 🡻 | - | (176) |
| **miR-340** | 🡹 | 🡹 | 🡻 | - | (177, 178) |
| **miR-370** | 🡹 | 🡻 | 🡻 | - | (179, 180) |
| **miR-382** |  | 🡻 | 🡻 | - | (181, 182) |
| **miR-467** | 🡹 |  |  | - | - |
| **miR-467c** | 🡹 |  |  | - | - |
| **miR-467d** | 🡹 |  |  | - | (183) |
| **miR-497** | 🡹 |  |  | (184, 185) | (186, 187) |
| **miR-503** |  | 🡻 | 🡻 | (188) | (189, 190) |
| **miR-540** |  |  | 🡹 | - | - |
| **miR-543** | 🡹 |  |  | (191) | (192, 193) |
| **miR-672** | 🡹 |  |  | - | - |
| **miR-674** | 🡹 | 🡻 | 🡻 | - | - |
| **miR-676** | 🡹 | 🡻 | 🡻 | - | - |
| **miR-680** | 🡹 | 🡹 | 🡻 | - | - |
| **miR-685** |  |  | 🡻 | - | - |
| **miR-690** |  | 🡻 | 🡻 | (194) | - |
| **miR-692** |  |  | 🡹 | - | - |
| **miR-698** |  | 🡹 | 🡹 | - | - |
| **miR-708** | 🡹 | 🡻 | 🡻 | (195) | (196, 197) |
| **miR-709** | 🡹 | 🡹 | 🡻 | (198) | (199) |
| **miR-720** |  |  | 🡻 | (200) | (201, 202) |
| **miR-721** | 🡻 | 🡹 | 🡹 | - | - |
| **miR-804** |  |  | 🡹 | - | - |
| **miR-805** | 🡹 |  |  | - | - |
| **miR-875-5p** | 🡹 |  |  | - | - |

Increase or decrease of miRNA expression over 2 fold is indicated in the table.

**Supplementary Table 2: p53 responsive elements in the promoter of miRNA upregulated in p53 wt cells.**

| **miRNA** | **Position**  **from-to** | **Matrix**  **similarity** | **Sequence**  **(red: ci-value > 60**  **CAPITALS: core sequence)** |
| --- | --- | --- | --- |
| **miR-298** | 735-757 | 0.805 | agggcCATGgtgtggcaagtatc |
|  | 808-830 | 0.737 | catctCATGgccagatctgtcta |
| **miR-467a1** | 596-618 | 0.942 | gaccctaggtatgtaCATGccca |
|  | 607-629 | 0.797 | cacaCAATatatgggcatgtaca |
| **miR-543** | 444-466 | 0.792 | agcaCAAGtacctgactaggaga |
|  | 1426-1448 | 0.827 | agcaCAAGtacctgactaggaga |
| **miR-709** | 540-562 | 0.789 | aataCACGtgtgggacaggcacc |

In silico analysis using Genomatix software revealed p53 responsive elements with high matrix similarity in several miRNA upregulated specifically in p53 wt cells.

**Supplementary Table 3: Predictive analysis identified responsive elements for miRNAs that are regulated by p53 in the 3’UTR of several genes involved in reprogramming and pluripotency .**

|  | **N-Myc** | **SSEA-1 /Fut4** | **Rex1** | **Akt1** | **Akt2** | **Akt3** | **ERK1** | **TRA-1-60 /PODXL** | **Smad2** | **Smad3** | **Oct3** |
| --- | --- | --- | --- | --- | --- | --- | --- | --- | --- | --- | --- |
| **miR-142-3p** |  |  | **1** |  |  | **2** |  | **1** |  |  |  |
| **miR-200c** |  |  |  |  | **1** | **5** |  |  | **2** | **1** | **2** |
| **miR-298** |  |  |  |  |  |  | **1** |  |  |  |  |
| **miR-467** | **1** |  |  | **1** | **1** |  |  |  | **1** |  |  |
| **miR-543** | **1** | **1** | **1** |  | **2** | **1** |  |  |  |  |  |
| **miR-709** | **1** | **2** |  | **1** | **2** | **1** |  |  |  |  |  |

The table indicates number of site present in the 3’UTR of the gene.

**References to Supplementary Table 1**

1. Foley NH, Bray I, Watters KM, Das S, Bryan K, Bernas T, et al. MicroRNAs 10a and 10b are potent inducers of neuroblastoma cell differentiation through targeting of nuclear receptor corepressor 2. Cell Death Differ. 2011 Jul;18(7):1089-98.

2. Huang H, Xie C, Sun X, Ritchie RP, Zhang J, Chen YE. miR-10a contributes to retinoid acid-induced smooth muscle cell differentiation. Journal Biol Chem. 2010 Mar 26;285(13):9383-9.

3. Li J, Dong J, Zhang ZH, Zhang DC, You XY, Zhong Y, et al. miR-10a restores human mesenchymal stem cell differentiation by repressing KLF4. J Cell Physiol. 2013 Dec;228(12):2324-36.

4. Li J, Zhang Y, Zhao Q, Wang J, He X. MicroRNA-10a Influences Osteoblast Differentiation and Angiogenesis by Regulating beta-Catenin Expression. Cellular physiology and biochemistry : international journal of experimental cellular physiology, biochemistry, and pharmacology. 2015;37(6):2194-208.

5. Agirre X, Jimenez-Velasco A, San Jose-Eneriz E, Garate L, Bandres E, Cordeu L, et al. Down-regulation of hsa-miR-10a in chronic myeloid leukemia CD34+ cells increases USF2-mediated cell growth. Molecular cancer research : MCR. 2008 Dec;6(12):1830-40.

6. Bryant A, Palma CA, Jayaswal V, Yang YW, Lutherborrow M, Ma DD. miR-10a is aberrantly overexpressed in Nucleophosmin1 mutated acute myeloid leukaemia and its suppression induces cell death. Molecular cancer. 2012;11:8.

7. Jia H, Zhang Z, Zou D, Wang B, Yan Y, Luo M, et al. MicroRNA-10a is down-regulated by DNA methylation and functions as a tumor suppressor in gastric cancer cells. PloS one. 2014;9(1):e88057.

8. Long MJ, Wu FX, Li P, Liu M, Li X, Tang H. MicroRNA-10a targets CHL1 and promotes cell growth, migration and invasion in human cervical cancer cells. Cancer letters. 2012 Nov 28;324(2):186-96.

9. Safari A, Seifoleslami M, Yahaghi E, Sedaghati F, Khameneie MK. Upregulation of miR-20a and miR-10a expression levels act as potential biomarkers of aggressive progression and poor prognosis in cervical cancer. Tumour biology : the journal of the International Society for Oncodevelopmental Biology and Medicine. 2015 Oct 1. P

10. Yan Y, Wang Q, Yan XL, Zhang Y, Li W, Tang F, et al. miR-10a controls glioma migration and invasion through regulating epithelial-mesenchymal transition via EphA8. FEBS letters. 2015 Mar 12;589(6):756-65.

11. Yu T, Liu L, Li J, Yan M, Lin H, Liu Y, et al. MiRNA-10a is upregulated in NSCLC and may promote cancer by targeting PTEN. Oncotarget. 2015 Oct 6;6(30):30239-50.

12. Vimalraj S, Partridge NC, Selvamurugan N. A positive role of microRNA-15b on regulation of osteoblast differentiation. Journal of cellular physiology. 2014 Sep;229(9):1236-44.

13. Li J, Chen Y, Guo X, Zhou L, Jia Z, Tang Y, et al. Inhibition of miR-15b decreases cell migration and metastasis in colorectal cancer. Tumour biology : the journal of the International Society for Oncodevelopmental Biology and Medicine. 2016 Jan 7.

14. Lovat F, Fassan M, Gasparini P, Rizzotto L, Cascione L, Pizzi M, et al. miR-15b/16-2 deletion promotes B-cell malignancies. Proceedings of the National Academy of Sciences of the United States of America. 2015 Sep 15;112(37):11636-41.

15. Sun L, Yao Y, Liu B, Lin Z, Lin L, Yang M, et al. MiR-200b and miR-15b regulate chemotherapy-induced epithelial-mesenchymal transition in human tongue cancer cells by targeting BMI1. Oncogene. 2012 Jan 26;31(4):432-45.

16. Xia H, Qi Y, Ng SS, Chen X, Chen S, Fang M, et al. MicroRNA-15b regulates cell cycle progression by targeting cyclins in glioma cells. Biochemical and biophysical research communications. 2009 Mar 6;380(2):205-10.

17. Zheng X, Chopp M, Lu Y, Buller B, Jiang F. MiR-15b and miR-152 reduce glioma cell invasion and angiogenesis via NRP-2 and MMP-3. Cancer letters. 2013 Feb 28;329(2):146-54.

18. Ben-Ami O, Pencovich N, Lotem J, Levanon D, Groner Y. A regulatory interplay between miR-27a and Runx1 during megakaryopoiesis. Proceedings of the National Academy of Sciences of the United States of America. 2009 Jan 6;106(1):238-43.

19. Guo D, Li Q, Lv Q, Wei Q, Cao S, Gu J. MiR-27a targets sFRP1 in hFOB cells to regulate proliferation, apoptosis and differentiation. PloS one. 2014;9(3):e91354.

20. Kim SY, Kim AY, Lee HW, Son YH, Lee GY, Lee JW, et al. miR-27a is a negative regulator of adipocyte differentiation via suppressing PPARgamma expression. Biochemical and biophysical research communications. 2010 Feb 12;392(3):323-8.

21. Ma Y, Yao N, Liu G, Dong L, Liu Y, Zhang M, et al. Functional screen reveals essential roles of miR-27a/24 in differentiation of embryonic stem cells. The EMBO journal. 2015 Feb 3;34(3):361-78.

22. Miao Y, Li J, Qiu X, Li Y, Wang Z, Luan Y. miR-27a regulates the self renewal of the H446 small cell lung cancer cell line in vitro. Oncology reports. 2013 Jan;29(1):161-8.

23. Min S, Li L, Zhang M, Zhang Y, Liang X, Xie Y, et al. TGF-beta-associated miR-27a inhibits dendritic cell-mediated differentiation of Th1 and Th17 cells by TAB3, p38 MAPK, MAP2K4 and MAP2K7. Genes and immunity. 2012 Dec;13(8):621-31.

24. Tang W, Yu F, Yao H, Cui X, Jiao Y, Lin L, et al. miR-27a regulates endothelial differentiation of breast cancer stem like cells. Oncogene. 2014 May 15;33(20):2629-38.

25. Colangelo T, Polcaro G, Ziccardi P, Muccillo L, Galgani M, Pucci B, et al. The miR-27a-calreticulin axis affects drug-induced immunogenic cell death in human colorectal cancer cells. Cell Death Dis. 2016;7:e2108.

26. Colangelo T, Polcaro G, Ziccardi P, Pucci B, Muccillo L, Galgani M, et al. Proteomic screening identifies calreticulin as a miR-27a direct target repressing MHC class I cell surface exposure in colorectal cancer. Cell Death Dis. 2016;7:e2120.

27. Liu T, Tang H, Lang Y, Liu M, Li X. MicroRNA-27a functions as an oncogene in gastric adenocarcinoma by targeting prohibitin. Cancer letters. 2009 Jan 18;273(2):233-42.

28. Mertens-Talcott SU, Chintharlapalli S, Li X, Safe S. The oncogenic microRNA-27a targets genes that regulate specificity protein transcription factors and the G2-M checkpoint in MDA-MB-231 breast cancer cells. Cancer research. 2007 Nov 15;67(22):11001-11.

29. Scheibner KA, Teaboldt B, Hauer MC, Chen X, Cherukuri S, Guo Y, et al. MiR-27a functions as a tumor suppressor in acute leukemia by regulating 14-3-3theta. PloS one. 2012;7(12):e50895.

30. Wang W, Cheng B, Miao L, Mei Y, Wu M. Mutant p53-R273H gains new function in sustained activation of EGFR signaling via suppressing miR-27a expression. Cell Death Dis. 2013;4:e574.

31. Zhang H, Li M, Han Y, Hong L, Gong T, Sun L, et al. Down-regulation of miR-27a might reverse multidrug resistance of esophageal squamous cell carcinoma. Digestive diseases and sciences. 2010 Sep;55(9):2545-51.

32. Zhang BW, Cai HF, Wei XF, Sun JJ, Lan XY, Lei CZ, et al. miR-30-5p Regulates Muscle Differentiation and Alternative Splicing of Muscle-Related Genes by Targeting MBNL. International journal of molecular sciences. 2016;17(2).

33. Baraniskin A, Birkenkamp-Demtroder K, Maghnouj A, Zollner H, Munding J, Klein-Scory S, et al. MiR-30a-5p suppresses tumor growth in colon carcinoma by targeting DTL. Carcinogenesis. 2012 Apr;33(4):732-9.

34. Wang X, Wang K, Han L, Zhang A, Shi Z, Zhang K, et al. PRDM1 is directly targeted by miR-30a-5p and modulates the Wnt/beta-catenin pathway in a Dkk1-dependent manner during glioma growth. Cancer letters. 2013 May 1;331(2):211-9.

35. Wang Z, Dai X, Chen Y, Sun C, Zhu Q, Zhao H, et al. MiR-30a-5p is induced by Wnt/beta-catenin pathway and promotes glioma cell invasion by repressing NCAM. Biochemical and biophysical research communications. 2015 Sep 25;465(3):374-80.

36. Xiong J, Wei B, Ye Q, Liu W. MiR-30a-5p/UBE3C axis regulates breast cancer cell proliferation and migration. Biochemical and biophysical research communications. 2016 Mar 18.

37. Karbiener M, Neuhold C, Opriessnig P, Prokesch A, Bogner-Strauss JG, Scheideler M. MicroRNA-30c promotes human adipocyte differentiation and co-represses PAI-1 and ALK2. RNA biology. 2011 Sep-Oct;8(5):850-60.

38. Huang J, Yao X, Zhang J, Dong B, Chen Q, Xue W, et al. Hypoxia-induced downregulation of miR-30c promotes epithelial-mesenchymal transition in human renal cell carcinoma. Cancer science. 2013 Dec;104(12):1609-17.

39. Tanic M, Yanowsky K, Rodriguez-Antona C, Andres R, Marquez-Rodas I, Osorio A, et al. Deregulated miRNAs in hereditary breast cancer revealed a role for miR-30c in regulating KRAS oncogene. PloS one. 2012;7(6):e38847.

40. Xia Y, Chen Q, Zhong Z, Xu C, Wu C, Liu B, et al. Down-regulation of miR-30c promotes the invasion of non-small cell lung cancer by targeting MTA1. Cellular physiology and biochemistry : international journal of experimental cellular physiology, biochemistry, and pharmacology. 2013;32(2):476-85.

41. Zhou H, Xu X, Xun Q, Yu D, Ling J, Guo F, et al. microRNA-30c negatively regulates endometrial cancer cells by targeting metastasis-associated gene-1. Oncology reports. 2012 Mar;27(3):807-12.

42. Lee H, Park CS, Deftereos G, Morihara J, Stern JE, Hawes SE, et al. MicroRNA expression in ovarian carcinoma and its correlation with clinicopathological features. World journal of surgical oncology. 2012;10:174.

43. Deng Y, Wu S, Zhou H, Bi X, Wang Y, Hu Y, et al. Effects of a miR-31, Runx2, and Satb2 regulatory loop on the osteogenic differentiation of bone mesenchymal stem cells. Stem cells and development. 2013 Aug 15;22(16):2278-86.

44. Weilner S, Schraml E, Wieser M, Messner P, Schneider K, Wassermann K, et al. Secreted microvesicular miR-31 inhibits osteogenic differentiation of mesenchymal stem cells. Aging cell. 2016 May 4.

45. Xie Q, Wang Z, Bi X, Zhou H, Wang Y, Gu P, et al. Effects of miR-31 on the osteogenesis of human mesenchymal stem cells. Biochemical and biophysical research communications. 2014 Mar 28;446(1):98-104.

46. Bhatnagar N, Li X, Padi SK, Zhang Q, Tang MS, Guo B. Downregulation of miR-205 and miR-31 confers resistance to chemotherapy-induced apoptosis in prostate cancer cells. Cell Death Dis. 2010;1:e105.

47. Ivanov SV, Goparaju CM, Lopez P, Zavadil J, Toren-Haritan G, Rosenwald S, et al. Pro-tumorigenic effects of miR-31 loss in mesothelioma. The Journal of biological chemistry. 2010 Jul 23;285(30):22809-17.

48. Liu CJ, Tsai MM, Hung PS, Kao SY, Liu TY, Wu KJ, et al. miR-31 ablates expression of the HIF regulatory factor FIH to activate the HIF pathway in head and neck carcinoma. Cancer research. 2010 Feb 15;70(4):1635-44.

49. Valastyan S, Chang A, Benaich N, Reinhardt F, Weinberg RA. Activation of miR-31 function in already-established metastases elicits metastatic regression. Genes & development. 2011 Mar 15;25(6):646-59.

50. Yamagishi M, Nakano K, Miyake A, Yamochi T, Kagami Y, Tsutsumi A, et al. Polycomb-mediated loss of miR-31 activates NIK-dependent NF-kappaB pathway in adult T cell leukemia and other cancers. Cancer cell. 2012 Jan 17;21(1):121-35.

51. Kuo PL, Liao SH, Hung JY, Huang MS, Hsu YL. MicroRNA-33a functions as a bone metastasis suppressor in lung cancer by targeting parathyroid hormone related protein. Biochimica et biophysica acta. 2013 Jun;1830(6):3756-66.

52. Thomas M, Lange-Grunweller K, Weirauch U, Gutsch D, Aigner A, Grunweller A, et al. The proto-oncogene Pim-1 is a target of miR-33a. Oncogene. 2012 Feb 16;31(7):918-28.

53. Yang L, Yang J, Li J, Shen X, Le Y, Zhou C, et al. MircoRNA-33a inhibits epithelial-to-mesenchymal transition and metastasis and could be a prognostic marker in non-small cell lung cancer. Scientific reports. 2015;5:13677.

54. Penzkofer D, Bonauer A, Fischer A, Tups A, Brandes RP, Zeiher AM, et al. Phenotypic characterization of miR-92a-/- mice reveals an important function of miR-92a in skeletal development. PloS one. 2014;9(6):e101153.

55. Wu Q, Yang Z, Wang F, Hu S, Yang L, Shi Y, et al. MiR-19b/20a/92a regulates the self-renewal and proliferation of gastric cancer stem cells. Journal of cell science. 2013 Sep 15;126(Pt 18):4220-9.

56. Yuva-Aydemir Y, Xu XL, Aydemir O, Gascon E, Sayin S, Zhou W, et al. Downregulation of the Host Gene jigr1 by miR-92 Is Essential for Neuroblast Self-Renewal in Drosophila. PLoS genetics. 2015 May;11(5):e1005264.

57. Chen ZL, Zhao XH, Wang JW, Li BZ, Wang Z, Sun J, et al. microRNA-92a promotes lymph node metastasis of human esophageal squamous cell carcinoma via E-cadherin. The Journal of biological chemistry. 2011 Mar 25;286(12):10725-34.

58. Nilsson S, Moller C, Jirstrom K, Lee A, Busch S, Lamb R, et al. Downregulation of miR-92a is associated with aggressive breast cancer features and increased tumour macrophage infiltration. PloS one. 2012;7(4):e36051.

59. Ren C, Wang W, Han C, Chen H, Fu D, Luo Y, et al. Expression and prognostic value of miR-92a in patients with gastric cancer. Tumour biology : the journal of the International Society for Oncodevelopmental Biology and Medicine. 2016 Jan 20.

60. Friedman JM, Liang G, Liu CC, Wolff EM, Tsai YC, Ye W, et al. The putative tumor suppressor microRNA-101 modulates the cancer epigenome by repressing the polycomb group protein EZH2. Cancer research. 2009 Mar 15;69(6):2623-9.

61. Wang HJ, Ruan HJ, He XJ, Ma YY, Jiang XT, Xia YJ, et al. MicroRNA-101 is down-regulated in gastric cancer and involved in cell migration and invasion. European journal of cancer (Oxford, England : 1990). 2010 Aug;46(12):2295-303.

62. Wang L, Zhang LF, Wu J, Xu SJ, Xu YY, Li D, et al. IL-1beta-mediated repression of microRNA-101 is crucial for inflammation-promoted lung tumorigenesis. Cancer research. 2014 Sep 1;74(17):4720-30.

63. Xiaoping L, Zhibin Y, Wenjuan L, Zeyou W, Gang X, Zhaohui L, et al. CPEB1, a histone-modified hypomethylated gene, is regulated by miR-101 and involved in cell senescence in glioma. Cell Death Dis. 2013;4:e675.

64. Yan D, Ng WL, Zhang X, Wang P, Zhang Z, Mo YY, et al. Targeting DNA-PKcs and ATM with miR-101 sensitizes tumors to radiation. PloS one. 2010;5(7):e11397.

65. Yan F, Shen N, Pang J, Xie D, Deng B, Molina JR, et al. Restoration of miR-101 suppresses lung tumorigenesis through inhibition of DNMT3a-dependent DNA methylation. Cell Death Dis. 2014;5:e1413.

66. Zhang X, Schulz R, Edmunds S, Kruger E, Markert E, Gaedcke J, et al. MicroRNA-101 Suppresses Tumor Cell Proliferation by Acting as an Endogenous Proteasome Inhibitor via Targeting the Proteasome Assembly Factor POMP. Molecular cell. 2015 Jul 16;59(2):243-57.

67. Huang F, Fang ZF, Hu XQ, Tang L, Zhou SH, Huang JP. Overexpression of miR-126 promotes the differentiation of mesenchymal stem cells toward endothelial cells via activation of PI3K/Akt and MAPK/ERK pathways and release of paracrine factors. Biological chemistry. 2013 Sep;394(9):1223-33.

68. Huang X, Gschweng E, Van Handel B, Cheng D, Mikkola HK, Witte ON. Regulated expression of microRNAs-126/126* inhibits erythropoiesis from human embryonic stem cells. Blood. 2011 Feb 17;117(7):2157-65.

69. Lechman ER, Gentner B, Ng SW, Schoof EM, van Galen P, Kennedy JA, et al. miR-126 Regulates Distinct Self-Renewal Outcomes in Normal and Malignant Hematopoietic Stem Cells. Cancer cell. 2016 Feb 8;29(2):214-28.

70. Okuyama K, Ikawa T, Gentner B, Hozumi K, Harnprasopwat R, Lu J, et al. MicroRNA-126-mediated control of cell fate in B-cell myeloid progenitors as a potential alternative to transcriptional factors. Proc. Nat. Acad. Sci. USA. 2013 Aug 13;110(33):13410-5.

71. Lechman ER, Gentner B, van Galen P, Giustacchini A, Saini M, Boccalatte FE, et al. Attenuation of miR-126 activity expands HSC in vivo without exhaustion. Cell stem cell. 2012 Dec 7;11(6):799-811.

72. Raffel S, Trumpp A. miR-126 Drives Quiescence and Self-Renewal in Leukemic Stem Cells. Cancer cell. 2016 Feb 8;29(2):133-5.

73. Crawford M, Brawner E, Batte K, Yu L, Hunter MG, Otterson GA, et al. MicroRNA-126 inhibits invasion in non-small cell lung carcinoma cell lines. Biochemical and biophysical research communications. 2008 Sep 5;373(4):607-12.

74. de Leeuw DC, Denkers F, Olthof MC, Rutten AP, Pouwels W, Schuurhuis GJ, et al. Attenuation of microRNA-126 expression that drives CD34+38- stem/progenitor cells in acute myeloid leukemia leads to tumor eradication. Cancer research. 2014 Apr 1;74(7):2094-105.

75. Donnem T, Lonvik K, Eklo K, Berg T, Sorbye SW, Al-Shibli K, et al. Independent and tissue-specific prognostic impact of miR-126 in nonsmall cell lung cancer: coexpression with vascular endothelial growth factor-A predicts poor survival. Cancer. 2011 Jul 15;117(14):3193-200.

76. Miko E, Margitai Z, Czimmerer Z, Varkonyi I, Dezso B, Lanyi A, et al. miR-126 inhibits proliferation of small cell lung cancer cells by targeting SLC7A5. FEBS letters. 2011 Apr 20;585(8):1191-6.

77. Zhang Y, Yang P, Sun T, Li D, Xu X, Rui Y, et al. miR-126 and miR-126* repress recruitment of mesenchymal stem cells and inflammatory monocytes to inhibit breast cancer metastasis. Nature cell biology. 2013 Mar;15(3):284-94.

78. Chen L, Wang GD, Liu JP, Wang HS, Liu XM, Wang Q, et al. miR-135a modulates tendon stem/progenitor cell senescence via suppressing ROCK1. Bone. 2015 Feb;71:210-6.

79. Moritoki Y, Hayashi Y, Mizuno K, Kamisawa H, Nishio H, Kurokawa S, et al. Expression profiling of microRNA in cryptorchid testes: miR-135a contributes to the maintenance of spermatogonial stem cells by regulating FoxO1. The Journal of urology. 2014 Apr;191(4):1174-80.

80. Holleman A, Chung I, Olsen RR, Kwak B, Mizokami A, Saijo N, et al. miR-135a contributes to paclitaxel resistance in tumor cells both in vitro and in vivo. Oncogene. 2011 Oct 27;30(43):4386-98.

81. Nagel R, le Sage C, Diosdado B, van der Waal M, Oude Vrielink JA, Bolijn A, et al. Regulation of the adenomatous polyposis coli gene by the miR-135 family in colorectal cancer. Cancer research. 2008 Jul 15;68(14):5795-802.

82. Navarro A, Diaz T, Martinez A, Gaya A, Pons A, Gel B, et al. Regulation of JAK2 by miR-135a: prognostic impact in classic Hodgkin lymphoma. Blood. 2009 Oct 1;114(14):2945-51.

83. Wu S, Lin Y, Xu D, Chen J, Shu M, Zhou Y, et al. MiR-135a functions as a selective killer of malignant glioma. Oncogene. 2012 Aug 23;31(34):3866-74.

84. Carraro G, Shrestha A, Rostkovius J, Contreras A, Chao CM, El Agha E, et al. miR-142-3p balances proliferation and differentiation of mesenchymal cells during lung development. Development (Cambridge, England). 2014 Mar;141(6):1272-81.

85. Lu X, Li X, He Q, Gao J, Gao Y, Liu B, et al. miR-142-3p regulates the formation and differentiation of hematopoietic stem cells in vertebrates. Cell research. 2013 Dec;23(12):1356-68.

86. Kwanhian W, Lenze D, Alles J, Motsch N, Barth S, Doll C, et al. MicroRNA-142 is mutated in about 20% of diffuse large B-cell lymphoma. Cancer medicine. 2012 Oct;1(2):141-55.

87. Lv M, Zhang X, Jia H, Li D, Zhang B, Zhang H, et al. An oncogenic role of miR-142-3p in human T-cell acute lymphoblastic leukemia (T-ALL) by targeting glucocorticoid receptor-alpha and cAMP/PKA pathways. Leukemia. 2012 Apr;26(4):769-77.

88. Xu G, Wang J, Jia Y, Shen F, Han W, Kang Y. MiR-142-3p functions as a potential tumor suppressor in human osteosarcoma by targeting HMGA1. Cellular physiology and biochemistry : international journal of experimental cellular physiology, biochemistry, and pharmacology. 2014;33(5):1329-39.

89. Song YX, Yue ZY, Wang ZN, Xu YY, Luo Y, Xu HM, et al. MicroRNA-148b is frequently down-regulated in gastric cancer and acts as a tumor suppressor by inhibiting cell proliferation. Molecular cancer. 2011;10:1.

90. Wang G, Cao X, Lai S, Luo X, Feng Y, Wu J, et al. Altered p53 regulation of miR-148b and p55PIK contributes to tumor progression in colorectal cancer. Oncogene. 2015 Feb 12;34(7):912-21.

91. Zhang JG, Shi Y, Hong DF, Song M, Huang D, Wang CY, et al. MiR-148b suppresses cell proliferation and invasion in hepatocellular carcinoma by targeting WNT1/beta-catenin pathway. Scientific reports. 2015;5:8087.

92. Zhao G, Zhang JG, Liu Y, Qin Q, Wang B, Tian K, et al. miR-148b functions as a tumor suppressor in pancreatic cancer by targeting AMPKalpha1. Molecular cancer therapeutics. 2013 Jan;12(1):83-93.

93. Fallah P, Arefian E, Naderi M, Aghaee-Bakhtiari SH, Atashi A, Ahmadi K, et al. miR-146a and miR-150 promote the differentiation of CD133+ cells into T-lymphoid lineage. Molecular biology reports. 2013 Aug;40(8):4713-9.

94. Smith NL, Wissink EM, Grimson A, Rudd BD. miR-150 Regulates Differentiation and Cytolytic Effector Function in CD8+ T cells. Scientific reports. 2015;5:16399.

95. Sun Z, Wang Y, Han X, Zhao X, Peng Y, Li Y, et al. miR-150 inhibits terminal erythroid proliferation and differentiation. Oncotarget. 2015 Dec 15;6(40):43033-47.

96. Warth SC, Hoefig KP, Hiekel A, Schallenberg S, Jovanovic K, Klein L, et al. Induced miR-99a expression represses Mtor cooperatively with miR-150 to promote regulatory T-cell differentiation. The EMBO journal. 2015 May 5;34(9):1195-213.

97. Xiao C, Calado DP, Galler G, Thai TH, Patterson HC, Wang J, et al. MiR-150 controls B cell differentiation by targeting the transcription factor c-Myb. Cell. 2007 Oct 5;131(1):146-59.

98. Aherne ST, Madden SF, Hughes DJ, Pardini B, Naccarati A, Levy M, et al. Circulating miRNAs miR-34a and miR-150 associated with colorectal cancer progression. BMC cancer. 2015;15:329.

99. Jiang X, Huang H, Li Z, Li Y, Wang X, Gurbuxani S, et al. Blockade of miR-150 maturation by MLL-fusion/MYC/LIN-28 is required for MLL-associated leukemia. Cancer cell. 2012 Oct 16;22(4):524-35.

100. Li YJ, Zhang YX, Wang PY, Chi YL, Zhang C, Ma Y, et al. Regression of A549 lung cancer tumors by anti-miR-150 vector. Oncology reports. 2012 Jan;27(1):129-34.

101. Yokobori T, Suzuki S, Tanaka N, Inose T, Sohda M, Sano A, et al. MiR-150 is associated with poor prognosis in esophageal squamous cell carcinoma via targeting the EMT inducer ZEB1. Cancer science. 2013 Jan;104(1):48-54.

102. Zhang N, Wei X, Xu L. miR-150 promotes the proliferation of lung cancer cells by targeting P53. FEBS letters. 2013 Aug 2;587(15):2346-51.

103. Liu F, Kong X, Lv L, Gao J. MiR-155 targets TP53INP1 to regulate liver cancer stem cell acquisition and self-renewal. FEBS letters. 2015 Feb 13;589(4):500-6.

104. Palma CA, Al Sheikha D, Lim TK, Bryant A, Vu TT, Jayaswal V, et al. MicroRNA-155 as an inducer of apoptosis and cell differentiation in Acute Myeloid Leukaemia. Molecular cancer. 2014;13:79.

105. Seok HY, Tatsuguchi M, Callis TE, He A, Pu WT, Wang DZ. miR-155 inhibits expression of the MEF2A protein to repress skeletal muscle differentiation. The Journal of biological chemistry. 2011 Oct 14;286(41):35339-46.

106. Eis PS, Tam W, Sun L, Chadburn A, Li Z, Gomez MF, et al. Accumulation of miR-155 and BIC RNA in human B cell lymphomas. Proceedings of the National Academy of Sciences of the United States of America. 2005 Mar 8;102(10):3627-32.

107. He XH, Zhu W, Yuan P, Jiang S, Li D, Zhang HW, et al. miR-155 downregulates ErbB2 and suppresses ErbB2-induced malignant transformation of breast epithelial cells. Oncogene. 2016 Apr 11.

108. Jiang S, Zhang HW, Lu MH, He XH, Li Y, Gu H, et al. MicroRNA-155 functions as an OncomiR in breast cancer by targeting the suppressor of cytokine signaling 1 gene. Cancer research. 2010 Apr 15;70(8):3119-27.

109. Tili E, Michaille JJ, Wernicke D, Alder H, Costinean S, Volinia S, et al. Mutator activity induced by microRNA-155 (miR-155) links inflammation and cancer. Proceedings of the National Academy of Sciences of the United States of America. 2011 Mar 22;108(12):4908-13. PubMed PMID: 21383199.

110. Kim KM, Park SJ, Jung SH, Kim EJ, Jogeswar G, Ajita J, et al. miR-182 is a negative regulator of osteoblast proliferation, differentiation, and skeletogenesis through targeting FoxO1. Journal of bone and mineral research : the official journal of the American Society for Bone and Mineral Research. 2012 Aug;27(8):1669-79.

111. Kouri FM, Hurley LA, Daniel WL, Day ES, Hua Y, Hao L, et al. miR-182 integrates apoptosis, growth, and differentiation programs in glioblastoma. Genes & development. 2015 Apr 1;29(7):732-45.

112. Guttilla IK, White BA. Coordinate regulation of FOXO1 by miR-27a, miR-96, and miR-182 in breast cancer cells. The Journal of biological chemistry. 2009 Aug 28;284(35):23204-16.

113. Li Y, Zhang D, Wang X, Yao X, Ye C, Zhang S, et al. Hypoxia-inducible miR-182 enhances HIF1alpha signaling via targeting PHD2 and FIH1 in prostate cancer. Scientific reports. 2015;5:12495.

114. Moskwa P, Buffa FM, Pan Y, Panchakshari R, Gottipati P, Muschel RJ, et al. miR-182-mediated downregulation of BRCA1 impacts DNA repair and sensitivity to PARP inhibitors. Molecular cell. 2011 Jan 21;41(2):210-20.

115. Segura MF, Hanniford D, Menendez S, Reavie L, Zou X, Alvarez-Diaz S, et al. Aberrant miR-182 expression promotes melanoma metastasis by repressing FOXO3 and microphthalmia-associated transcription factor. Proceedings of the National Academy of Sciences of the United States of America. 2009 Feb 10;106(6):1814-9.

116. Wang J, Li J, Shen J, Wang C, Yang L, Zhang X. MicroRNA-182 downregulates metastasis suppressor 1 and contributes to metastasis of hepatocellular carcinoma. BMC cancer. 2012;12:227.

117. Zhang Y, Wang X, Wang Z, Tang H, Fan H, Guo Q. miR-182 promotes cell growth and invasion by targeting forkhead box F2 transcription factor in colorectal cancer. Oncology reports. 2015 May;33(5):2592-8.

118. Cai J, Wu J, Zhang H, Fang L, Huang Y, Yang Y, et al. miR-186 downregulation correlates with poor survival in lung adenocarcinoma, where it interferes with cell-cycle regulation. Cancer research. 2013 Jan 15;73(2):756-66.

119. Zhou L, Qi X, Potashkin JA, Abdul-Karim FW, Gorodeski GI. MicroRNAs miR-186 and miR-150 down-regulate expression of the pro-apoptotic purinergic P2X7 receptor by activation of instability sites at the 3'-untranslated region of the gene that decrease steady-state levels of the transcript. The Journal of biological chemistry. 2008 Oct 17;283(42):28274-86.

120. Zhu X, Shen H, Yin X, Long L, Xie C, Liu Y, et al. miR-186 regulation of Twist1 and ovarian cancer sensitivity to cisplatin. Oncogene. 2016 Jan 21;35(3):323-32.

121. Jeong BC, Kang IH, Hwang YC, Kim SH, Koh JT. MicroRNA-194 reciprocally stimulates osteogenesis and inhibits adipogenesis via regulating COUP-TFII expression. Cell death & disease. 2014;5:e1532.

122. Li J, He X, Wei W, Zhou X. MicroRNA-194 promotes osteoblast differentiation via downregulating STAT1. Biochemical and biophysical research communications. 2015 May 1;460(2):482-8.

123. Xu J, Kang Y, Liao WM, Yu L. MiR-194 regulates chondrogenic differentiation of human adipose-derived stem cells by targeting Sox5. PloS one. 2012;7(3):e31861.

124. Sundaram P, Hultine S, Smith LM, Dews M, Fox JL, Biyashev D, et al. p53-responsive miR-194 inhibits thrombospondin-1 and promotes angiogenesis in colon cancers. Cancer research. 2011 Dec 15;71(24):7490-501.

125. Wong TS, Liu XB, Wong BY, Ng RW, Yuen AP, Wei WI. Mature miR-184 as Potential Oncogenic microRNA of Squamous Cell Carcinoma of Tongue. Clinical cancer research : an official journal of the American Association for Cancer Research. 2008 May 1;14(9):2588-92.

126. Wu X, Liu T, Fang O, Leach LJ, Hu X, Luo Z. miR-194 suppresses metastasis of non-small cell lung cancer through regulating expression of BMP1 and p27(kip1). Oncogene. 2014 Mar 20;33(12):1506-14.

127. Zhu X, Li D, Yu F, Jia C, Xie J, Ma Y, et al. miR-194 inhibits the proliferation, invasion, migration, and enhances the chemosensitivity of non-small cell lung cancer cells by targeting forkhead box A1 protein. Oncotarget. 2016 Mar 15;7(11):13139-52.

128. Chen T, Margariti A, Kelaini S, Cochrane A, Guha ST, Hu Y, et al. MicroRNA-199b Modulates Vascular Cell Fate During iPS Cell Differentiation by Targeting the Notch Ligand Jagged1 and Enhancing VEGF Signaling. Stem cells (Dayton, Ohio). 2015 May;33(5):1405-18.

129. Chao A, Tsai CL, Wei PC, Hsueh S, Chao AS, Wang CJ, et al. Decreased expression of microRNA-199b increases protein levels of SET (protein phosphatase 2A inhibitor) in human choriocarcinoma. Cancer letters. 2010 May 1;291(1):99-107.

130. Shen ZL, Wang B, Jiang KW, Ye CX, Cheng C, Yan YC, et al. Downregulation of miR-199b is associated with distant metastasis in colorectal cancer via activation of SIRT1 and inhibition of CREB/KISS1 signaling. Oncotarget. 2016 Apr 27.

131. Eggers JC, Martino V, Reinbold R, Schafer SD, Kiesel L, Starzinski-Powitz A, et al. microRNA miR-200b affects proliferation, invasiveness and stemness of endometriotic cells by targeting ZEB1, ZEB2 and KLF4. Reproductive biomedicine online. 2016 Apr;32(4):434-45.

132. Bracken CP, Li X, Wright JA, Lawrence DM, Pillman KA, Salmanidis M, et al. Genome-wide identification of miR-200 targets reveals a regulatory network controlling cell invasion. The EMBO journal. 2014 Sep 17;33(18):2040-56.

133. Gregory PA, Bert AG, Paterson EL, Barry SC, Tsykin A, Farshid G, et al. The miR-200 family and miR-205 regulate epithelial to mesenchymal transition by targeting ZEB1 and SIP1. Nature cell biology. 2008 May;10(5):593-601.

134. Kim Y, Park D, Kim H, Choi M, Lee H, Lee YS, et al. miR-200b and cancer/testis antigen CAGE form a feedback loop to regulate the invasion and tumorigenic and angiogenic responses of a cancer cell line to microtubule-targeting drugs. The Journal of biological chemistry. 2013 Dec 20;288(51):36502-18.

135. Tellez CS, Juri DE, Do K, Bernauer AM, Thomas CL, Damiani LA, et al. EMT and stem cell-like properties associated with miR-205 and miR-200 epigenetic silencing are early manifestations during carcinogen-induced transformation of human lung epithelial cells. Cancer research. 2011 Apr 15;71(8):3087-97.

136. Wu H, Wang G, Wang Z, An S, Ye P, Luo S. A negative feedback loop between miR-200b and the NF-kappaB pathway via IKBKB/IKK-beta in breast cancer cells. The FEBS journal. 2015 Oct 3.

137. Xu CX, Xu M, Tan L, Yang H, Permuth-Wey J, Kruk PA, et al. MicroRNA miR-214 regulates ovarian cancer cell stemness by targeting p53/Nanog. The Journal of biological chemistry. 2012 Oct 12;287(42):34970-8.

138. Chang CJ, Chao CH, Xia W, Yang JY, Xiong Y, Li CW, et al. p53 regulates epithelial-mesenchymal transition and stem cell properties through modulating miRNAs. Nature cell biology. 2011 Mar;13(3):317-23.

139. Huang J, Zhao L, Xing L, Chen D. MicroRNA-204 regulates Runx2 protein expression and mesenchymal progenitor cell differentiation. Stem cells (Dayton, Ohio). 2010 Feb;28(2):357-64.

140. Hall DP, Cost NG, Hegde S, Kellner E, Mikhaylova O, Stratton Y, et al. TRPM3 and miR-204 establish a regulatory circuit that controls oncogenic autophagy in clear cell renal cell carcinoma. Cancer cell. 2014 Nov 10;26(5):738-53.

141. Kuwano Y, Nishida K, Kajita K, Satake Y, Akaike Y, Fujita K, et al. Transformer 2beta and miR-204 regulate apoptosis through competitive binding to 3' UTR of BCL2 mRNA. Cell death and differentiation. 2015 May;22(5):815-25.

142. Lee H, Lee S, Bae H, Kang HS, Kim SJ. Genome-wide identification of target genes for miR-204 and miR-211 identifies their proliferation stimulatory role in breast cancer cells. Scientific reports. 2016;6:25287.

143. Sacconi A, Biagioni F, Canu V, Mori F, Di Benedetto A, Lorenzon L, et al. miR-204 targets Bcl-2 expression and enhances responsiveness of gastric cancer. C Cell Death Dis. 2012;3:e423.

144. Wu X, Zeng Y, Wu S, Zhong J, Wang Y, Xu J. MiR-204, down-regulated in retinoblastoma, regulates proliferation and invasion of human retinoblastoma cells by targeting CyclinD2 and MMP-9. FEBS letters. 2015 Feb 27;589(5):645-50.

145. Ying Z, Li Y, Wu J, Zhu X, Yang Y, Tian H, et al. Loss of miR-204 expression enhances glioma migration and stem cell-like phenotype. Cancer research. 2013 Jan 15;73(2):990-9.

146. Dey BK, Gagan J, Dutta A. miR-206 and -486 induce myoblast differentiation by downregulating Pax7. Molecular and cellular biology. 2011 Jan;31(1):203-14.

147. Kim HK, Lee YS, Sivaprasad U, Malhotra A, Dutta A. Muscle-specific microRNA miR-206 promotes muscle differentiation. The Journal of cell biology. 2006 Aug 28;174(5):677-87.

148. Kondo N, Toyama T, Sugiura H, Fujii Y, Yamashita H. miR-206 Expression is down-regulated in estrogen receptor alpha-positive human breast cancer. Cancer research. 2008 Jul 1;68(13):5004-8.

149. Miyachi M, Tsuchiya K, Yoshida H, Yagyu S, Kikuchi K, Misawa A, et al. Circulating muscle-specific microRNA, miR-206, as a potential diagnostic marker for rhabdomyosarcoma. Biochemical and biophysical research communications. 2010 Sep 10;400(1):89-93.

150. Gay I, Cavender A, Peto D, Sun Z, Speer A, Cao H, et al. Differentiation of human dental stem cells reveals a role for microRNA-218. Journal of periodontal research. 2014 Feb;49(1):110-20.

151. Hu K, Xu C, Ni H, Xu Z, Wang Y, Xu S, et al. Mir-218 contributes to the transformation of 5-Aza/GF induced umbilical cord mesenchymal stem cells into hematopoietic cells through the MITF pathway. Molecular biology reports. 2014 Jul;41(7):4803-16.

152. Zhang WB, Zhong WJ, Wang L. A signal-amplification circuit between miR-218 and Wnt/beta-catenin signal promotes human adipose tissue-derived stem cells osteogenic differentiation. Bone. 2014 Jan;58:59-66.

153. Cheng Y, Yang X, Deng X, Zhang X, Li P, Tao J, et al. MicroRNA-218 inhibits bladder cancer cell proliferation, migration, and invasion by targeting BMI-1. Tumour biology : the journal of the International Society for Oncodevelopmental Biology and Medicine. 2015 Sep;36(10):8015-23.

154. Zhu K, Ding H, Wang W, Liao Z, Fu Z, Hong Y, et al. Tumor-suppressive miR-218-5p inhibits cancer cell proliferation and migration via EGFR in non-small cell lung cancer. Oncotarget. 2016 Apr 4.

155. Sher YP, Wang LJ, Chuang LL, Tsai MH, Kuo TT, Huang CC, et al. ADAM9 up-regulates N-cadherin via miR-218 suppression in lung adenocarcinoma cells. PloS one. 2014;9(4):e94065.

156. Goldberger N, Walker RC, Kim CH, Winter S, Hunter KW. Inherited variation in miR-290 expression suppresses breast cancer progression by targeting the metastasis susceptibility gene Arid4b. Cancer research. 2013 Apr 15;73(8):2671-81.

157. Guo WT, Wang XW, Yan YL, Li YP, Yin X, Zhang Q, et al. Suppression of epithelial-mesenchymal transition and apoptotic pathways by miR-294/302 family synergistically blocks let-7-induced silencing of self-renewal in embryonic stem cells. Cell death and differentiation. 2015 Jul;22(7):1158-69.

158. Wang Y, Melton C, Li YP, Shenoy A, Zhang XX, Subramanyam D, et al. miR-294/miR-302 promotes proliferation, suppresses G1-S restriction point, and inhibits ESC differentiation through separable mechanisms. Cell reports. 2013 Jul 11;4(1):99-109.

159. Vaira V, Faversani A, Dohi T, Montorsi M, Augello C, Gatti S, et al. miR-296 regulation of a cell polarity-cell plasticity module controls tumor progression. Oncogene. 2012 Jan 5;31(1):27-38.

160. Wurdinger T, Tannous BA, Saydam O, Skog J, Grau S, Soutschek J, et al. miR-296 regulates growth factor receptor overexpression in angiogenic endothelial cells. Cancer cell. 2008 Nov 4;14(5):382-93.

161. Bai Y, Liao H, Liu T, Zeng X, Xiao F, Luo L, et al. MiR-296-3p regulates cell growth and multi-drug resistance of human glioblastoma by targeting ether-a-go-go (EAG1). European journal of cancer (Oxford, England : 1990). 2013 Feb;49(3):710-24.

162. Liu X, Chen Q, Yan J, Wang Y, Zhu C, Chen C, et al. MiRNA-296-3p-ICAM-1 axis promotes metastasis of prostate cancer by possible enhancing survival of natural killer cell-resistant circulating tumour cells. Cell Death Dis. 2013;4:e928.

163. Bao L, Hazari S, Mehra S, Kaushal D, Moroz K, Dash S. Increased expression of P-glycoprotein and doxorubicin chemoresistance of metastatic breast cancer is regulated by miR-298. The American journal of pathology. 2012 Jun;180(6):2490-503.

164. Shi W, Gerster K, Alajez NM, Tsang J, Waldron L, Pintilie M, et al. MicroRNA-301 mediates proliferation and invasion in human breast cancer. Cancer research. 2011 Apr 15;71(8):2926-37.

165. Egawa H, Jingushi K, Hirono T, Ueda Y, Kitae K, Nakata W, et al. The miR-130 family promotes cell migration and invasion in bladder cancer through FAK and Akt phosphorylation by regulating PTEN. Scientific reports. 2016;6:20574.

166. Funamizu N, Lacy CR, Parpart ST, Takai A, Hiyoshi Y, Yanaga K. MicroRNA-301b promotes cell invasiveness through targeting TP63 in pancreatic carcinoma cells. International journal of oncology. 2014 Mar;44(3):725-34.

167. Guo YJ, Liu JX, Guan YW. Hypoxia induced upregulation of miR-301a/b contributes to increased cell autophagy and viability of prostate cancer cells by targeting NDRG2. European review for medical and pharmacological sciences. 2016 Jan;20(1):101-8.

168. Zhang Y, Teng F, Luo GZ, Wang M, Tong M, Zhao X, et al. MicroRNA-323-3p regulates the activity of polycomb repressive complex 2 (PRC2) via targeting the mRNA of embryonic ectoderm development (Eed) gene in mouse embryonic stem cells. The Journal of biological chemistry. 2013 Aug 16;288(33):23659-65.

169. Wang C, Liu P, Wu H, Cui P, Li Y, Liu Y, et al. MicroRNA-323-3p inhibits cell invasion and metastasis in pancreatic ductal adenocarcinoma via direct suppression of SMAD2 and SMAD3. Oncotarget. 2016 Feb 18.

170. Xu J, Ai Q, Cao H, Liu Q. MiR-185-3p and miR-324-3p Predict Radiosensitivity of Nasopharyngeal Carcinoma and Modulate Cancer Cell Growth and Apoptosis by Targeting SMAD7. Medical science monitor : international medical journal of experimental and clinical research. 2015;21:2828-36.

171. Feng DD, Zhang H, Zhang P, Zheng YS, Zhang XJ, Han BW, et al. Down-regulated miR-331-5p and miR-27a are associated with chemotherapy resistance and relapse in leukaemia. Journal of cellular and molecular medicine. 2011 Oct;15(10):2164-75.

172. Schoeftner S, Scarola M, Comisso E, Schneider C, Benetti R. An Oct4-pRb axis, controlled by MiR-335, integrates stem cell self-renewal and cell cycle control. Stem cells (Dayton, Ohio). 2013 Apr;31(4):717-28.

173. Tome M, Lopez-Romero P, Albo C, Sepulveda JC, Fernandez-Gutierrez B, Dopazo A, et al. miR-335 orchestrates cell proliferation, migration and differentiation in human mesenchymal stem cells. Cell death and differentiation. 2011 Jun;18(6):985-95.

174. Png KJ, Yoshida M, Zhang XH, Shu W, Lee H, Rimner A, et al. MicroRNA-335 inhibits tumor reinitiation and is silenced through genetic and epigenetic mechanisms in human breast cancer. Genes & development. 2011 Feb 1;25(3):226-31.

175. Scarola M, Schoeftner S, Schneider C, Benetti R. miR-335 directly targets Rb1 (pRb/p105) in a proximal connection to p53-dependent stress response. Cancer research. 2010 Sep 1;70(17):6925-33.

176. Zhou C, Lu Y, Li X. miR-339-3p inhibits proliferation and metastasis of colorectal cancer. Oncology letters. 2015 Nov;10(5):2842-8.

177. Li X, Gong X, Chen J, Zhang J, Sun J, Guo M. miR-340 inhibits glioblastoma cell proliferation by suppressing CDK6, cyclin-D1 and cyclin-D2. Biochemical and biophysical research communications. 2015 May 8;460(3):670-7.

178. Wu ZS, Wu Q, Wang CQ, Wang XN, Huang J, Zhao JJ, et al. miR-340 inhibition of breast cancer cell migration and invasion through targeting of oncoprotein c-Met. Cancer. 2011 Jul 1;117(13):2842-52.

179. Lo SS, Hung PS, Chen JH, Tu HF, Fang WL, Chen CY, et al. Overexpression of miR-370 and downregulation of its novel target TGFbeta-RII contribute to the progression of gastric carcinoma. Oncogene. 2012 Jan 12;31(2):226-37.

180. Wu Z, Sun H, Zeng W, He J, Mao X. Upregulation of MircoRNA-370 induces proliferation in human prostate cancer cells by downregulating the transcription factor FOXO1. PloS one. 2012;7(9):e45825.

181. Seok JK, Lee SH, Kim MJ, Lee YM. MicroRNA-382 induced by HIF-1alpha is an angiogenic miR targeting the tumor suppressor phosphatase and tensin homolog. Nucleic acids research. 2014 Jul;42(12):8062-72.

182. Xu M, Jin H, Xu CX, Sun B, Mao Z, Bi WZ, et al. miR-382 inhibits tumor growth and enhance chemosensitivity in osteosarcoma. Oncotarget. 2014 Oct 15;5(19):9472-83.

183. Necela BM, Carr JM, Asmann YW, Thompson EA. Differential expression of microRNAs in tumors from chronically inflamed or genetic (APC(Min/+)) models of colon cancer. PloS one. 2011;6(4):e18501.

184. Grunhagen J, Bhushan R, Degenkolbe E, Jager M, Knaus P, Mundlos S, et al. MiR-497 approximately 195 cluster microRNAs regulate osteoblast differentiation by targeting BMP signaling. Journal of bone and mineral research : the official journal of the American Society for Bone and Mineral Research. 2015 May;30(5):796-808.

185. Sato T, Yamamoto T, Sehara-Fujisawa A. miR-195/497 induce postnatal quiescence of skeletal muscle stem cells. Nature communications. 2014;5:4597.

186. Guo ST, Jiang CC, Wang GP, Li YP, Wang CY, Guo XY, et al. MicroRNA-497 targets insulin-like growth factor 1 receptor and has a tumour suppressive role in human colorectal cancer. Oncogene. 2013 Apr 11;32(15):1910-20.

187. Lan J, Xue Y, Chen H, Zhao S, Wu Z, Fang J, et al. Hypoxia-induced miR-497 decreases glioma cell sensitivity to TMZ by inhibiting apoptosis. FEBS letters. 2014 Sep 17;588(18):3333-9.

188. Sarkar S, Dey BK, Dutta A. MiR-322/424 and -503 are induced during muscle differentiation and promote cell cycle quiescence and differentiation by down-regulation of Cdc25A. Molecular biology of the cell. 2010 Jul 1;21(13):2138-49.

189. Xu YY, Wu HJ, Ma HD, Xu LP, Huo Y, Yin LR. MicroRNA-503 suppresses proliferation and cell-cycle progression of endometrioid endometrial cancer by negatively regulating cyclin D1. The FEBS journal. 2013 Aug;280(16):3768-79.

190. Zhou J, Wang W. Analysis of microRNA expression profiling identifies microRNA-503 regulates metastatic function in hepatocellular cancer cell. Journal of surgical oncology. 2011 Sep 1;104(3):278-83.

191. Lee S, Yu KR, Ryu YS, Oh YS, Hong IS, Kim HS, et al. miR-543 and miR-590-3p regulate human mesenchymal stem cell aging via direct targeting of AIMP3/p18. Age (Dordrecht, Netherlands). 2014;36(6):9724.

192. Bing L, Hong C, Li-Xin S, Wei G. MicroRNA-543 suppresses endometrial cancer oncogenicity via targeting FAK and TWIST1 expression. Archives of gynecology and obstetrics. 2014 Sep;290(3):533-41.

193. Fan C, Lin Y, Mao Y, Huang Z, Liu AY, Ma H, et al. MicroRNA-543 suppresses colorectal cancer growth and metastasis by targeting KRAS, MTA1 and HMGA2. Oncotarget. 2016 Mar 8.

194. Yu S, Geng Q, Pan Q, Liu Z, Ding S, Xiang Q, et al. MiR-690, a Runx2-targeted miRNA, regulates osteogenic differentiation of C2C12 myogenic progenitor cells by targeting NF-kappaB p65. Cell & bioscience. 2016;6:10.

195. Hao C, Yang S, Xu W, Shen JK, Ye S, Liu X, et al. MiR-708 promotes steroid-induced osteonecrosis of femoral head, suppresses osteogenic differentiation by targeting SMAD3. Scientific reports. 2016;6:22599.

196. Guo P, Lan J, Ge J, Nie Q, Mao Q, Qiu Y. miR-708 acts as a tumor suppressor in human glioblastoma cells. Oncology reports. 2013 Aug;30(2):870-6.

197. Saini S, Yamamura S, Majid S, Shahryari V, Hirata H, Tanaka Y, et al. MicroRNA-708 induces apoptosis and suppresses tumorigenicity in renal cancer cells. Cancer research. 2011 Oct 1;71(19):6208-19.

198. Chen H, Mo D, Li M, Zhang Y, Chen L, Zhang X, et al. miR-709 inhibits 3T3-L1 cell differentiation by targeting GSK3beta of Wnt/beta-catenin signaling. Cellular signalling. 2014 Nov;26(11):2583-9.

199. Liu T, Zhang X, Sha K, Liu X, Zhang L, Wang B. miR-709 up-regulated in hepatocellular carcinoma, promotes proliferation and invasion by targeting GPC5. Cell proliferation. 2015 Jun;48(3):330-7.

200. Hara ES, Ono M, Eguchi T, Kubota S, Pham HT, Sonoyama W, et al. miRNA-720 controls stem cell phenotype, proliferation and differentiation of human dental pulp cells. PloS one. 2013;8(12):e83545.

201. Das SG, Romagnoli M, Mineva ND, Barille-Nion S, Jezequel P, Campone M, et al. miR-720 is a downstream target of an ADAM8-induced ERK signaling cascade that promotes the migratory and invasive phenotype of triple-negative breast cancer cells. Breast cancer research : BCR. 2016;18(1):40.

202. Li LZ, Zhang CZ, Liu LL, Yi C, Lu SX, Zhou X, et al. miR-720 inhibits tumor invasion and migration in breast cancer by targeting TWIST1. Carcinogenesis. 2014 Feb;35(2):469-78.
